# Supplementary material for: Bran-Enriched Milled Durum Wheat Fractions Obtained Using Innovative Micronization and Air-Classification Pilot Plants
Source: Foods. 2021 Aug 3;10(8):1796. doi: 10.3390/foods10081796 (PMC8391628; doi:10.3390/foods10081796)
Supplement: Supplementary file 1 [file foods-10-01796-s001.zip › foods-1283703-supplementary.pdf]

Supplementary Materials:

Table S1. Mean yield percentage (%± S.D.) of micronized samples and air-classified fractions (cv. Saragolla, Maestà and Iride). Same letter: no significant difference (p>0.05) within all fractions (F and G types) for each cultivar (n=2).

	Air classified fractions													
**Cultivar**	**F220**	**F230**	**F240**	**F250**	**F260**	**F270**	**F280**	**G220**	**G230**	**G240**	**G250**	**G260**	**G270**	**G280**
**Saragolla**	20.8±0.3^{m}	32.2±2.8^{i}	44.6±1.3^{h}	71.5±0.5^{e}	83.1±0.1^{c}	90.9±0.5^{bc}	93.6±0.1^{ab}	79.0±0.4^{cde}	67.7±2.8^{ef}	55.3±1.5^{g}	27.9±0.4^{ilm}	16.5±0.3^{mn}	9.1±0.6^{no}	5.8±0.1^{o}
**Maestà**	27.5±5.9^{m}	35.2±4.8^{im}	45.5±0.8^{him}	75.4±5.4^{e}	89.8±1.1^{ce}	92.2±0.3bce	94.4±0.6^{abce}	72.1±6.0^{abcde}	64.5±4.5^{defh}	54.1±0.6^{defghi}	24.4±5.2^{hilm}	9.3±0.4^{lmn}	7.5±0.4^{lmn}	5.3±0.8^{mn}
**Iride**	21.1±0.6^{m}	33.8±0.4^{i}	53.9±0.6^{h}	75.2±1.3^{e}	84.4±2.5^{c}	90.5±0.6bc	92.0±0.3^{abc}	78.5±0.6^{cde}	65.5±0.4^{f}	46.1±0.6^{g}	24.8±1.3^{lm}	15.5±2.5^{mn}	9.4±0.4^{n}	8.0±0.3^{n}

Table S2. Mean ash content (% dry basis ± S.D.) of micronized samples and air-classified fractions (cv. Saragolla, Maestà and Iride). Same letter: no significant difference (p>0.05) within all fractions (F and G types) for each cultivar (n=2).

		Air classified fractions													
**Cultivar**	**Micronized**	**F220**	**F230**	**F240**	**F250**	**F260**	**F270**	**F280**	**G220**	**G230**	**G240**	**G250**	**G260**	**G270**	**G280**
**Saragolla**	1.97±0.00	3.10±0.06^{a}	2.68±0.19^{ab}	2.38±0.00^{bc}	2.19±0.10^{bcd}	2.12±0.17^{bcde}	2.24±0.10^{bcdef}	1.97±0.01^{cdefg}	1.72±0.08^{defg}	1.60±0.09^{defg}	1.57±0.01^{deg}	1.50±0.11^{eg}	1.39±0.08^{g}	1.56±0.00^{deg}	1.32±0.05^{g}
**Maestà**	2.15±0.04	3.38±0.25^{a}	3.09±0.06^{ab}	2.79±0.00^{abc}	2.42±0.00^{cd}	2.19±0.13^{cde}	2.24±0.10^{cdef}	2.24±0.05^{cdefg}	1.77±0.11^{defgh}	1.72±0.08^{efgh}	1.67±0.00^{efgh}	1.64±0.02^{efgh}	1.58±0.08^{efgh}	1.56±0.07^{eh}	1.55±0.00^{eh}
**Iride**	1.97±0.00	2.99±0.07^{a}	2.47±0.10^{b}	2.15±0.02^{bc}	1.98±0.05^{cd}	1.99±0.00^{cde}	1.88±0.05^{cdef}	1.91±0.01^{cdefg}	1.51±0.10^{fgh}	1.55±0.16^{defgh}	1.32±0.04^{h}	1.64±0.05^{defgh}	1.65±0.01^{defgh}	1.53±0.01^{dfgh}	1.38±0.02^{h}

Table S3. Mean particle size content (% ± Standard Deviation) of micronized samples and air-classified fractions (cv. Saragolla, Maestà and Iride). Same letter: no significant difference (p>0.05) within all fractions (F and G types) for each cultivar (n=2).

Air classified fraction Micronized samples and air-classified fractions (particle size: ø > 425 µm)

Cultivar	Micronized	F220	F230	F240	F250	F260	F270	F280	G220	G230	G240	G250	G260	G270	G280
Saragolla	9.73±0.06	38.29±3.44^{b}	6.22±0.57^{c}	6.32±1.23^{ac}	5.10±0.14^{ac}	6.09±0.33^{ac}	10.25±3.47^{abc}	8.96±2.56^{abc}	12.45±1.96^{abc}	12.22±2.17^{abc}	13.98±0.42^{abc}	23.85±0.91^{ab}	32.20±3.01^{ab}	50.28±3.09^{ab}	51.25±2.9^{8} ^{ab}
Maestà	10.68±0.11	11.78±0.46^{b}	7.85±0.97^{bc}	5.24±0.10^{bcd}	3.95±0.77^{ed}	6.95±0.78^{bcde}	6.09±0.88^{bcde}	7.66±0.61^{abcd}	12.96±0.65^{abcd}	12.39±1.63^{abcd}	15.31±1.12^{abc}	27.71±2.26^{abc}	45.22±0.62^{abc}	50.85±2.07^{abc}	56.61±1.39^{abc}
Iride	11.38±0.40	9.50±0.17^{b}	5.66±0.12^{b}	4.91±0.42^{b}	3.98±0.67^{b}	6.32±0.18^{b}	7.03±0.45^{b}	7.49±0.67^{bc}	8.87±2.28^{abc}	11.89±1.30^{abc}	13.14±0.71^{abc}	32.46±2.77^{abc}	40.08±1.54^{abc}	48.68±1.11^{ab}	52.31±2.15^{ab}

Micronized samples and air-classified fractions (particle size: 425 µm > ø > 180µm)

Cultivar	Micronized	F220	F230	F240	F250	F260	F270	F280	G220	G230	G240	G250	G260	G270	G280
Saragolla	43.66±1.84	46.29±0.69^{l}	61.39±2.96^{c}	46.97±1.20^{hl}	47.25±0.22^{ghl}	50.22±1.15^{ceghl}	46.91±2.69^{eghil}	53.30±1.34^{cdeghil}	48.12±1.51^{cdefghil}	55.43±0.55^{cdefghil}	63.09±0.32^{bcde}	72.95±0.24^{a}	65.23±0.57^{abcd}	49.43±3.00^{bcdfeghil}	48.52±2.84^{cd} ^{eghil}
Maestà	49.95±1.13	68.37±2.22^{b}	54.28±2.16^{bf}	41.07±2.01^{n}	48.55±0.41^{fmn}	52.26±0.46^{fim}	52.36±0.58^{fhim}	53.21±0.97^{fghim}	51.50±1.31^{fghilm}	56.51±0.29^{bdfghilm}	64.51±1.98^{bcdfghi}	70.33±1.51^{a}	54.28±0.54^{bcdeglm}	48.82±1.92^{defghilm}	43.19±1.32^{lmn}
Iride	44.92±0.53	69.86±1.32^{b}	49.86±1.80^{i}	38.13±0.24^{n}	41.56±0.65^{imn}	50.59±0.13^{hi}	52.73±2.77^{fhi}	51.93±1.85^{fghi}	46.66±2.12^{fghilm}	55.98±1.40^{befghil}	71.14±1.06^{abe}	66.17±2.67^{alfg}	59.37±1.48^{abcdhi}	50.90±1.09^{defghil}	47.41±2.26^{efg} ^{him}

Micronized samples and air-classified fractions (particle size: 180µm > ø)

Cultivar	Micronized	F220	F230	F240	F250	F260	F270	F280	G220	G230	G240	G250	G260	G270	G280
Saragol	46.60±1.78	15.81±2.75^{h}	32.40±3.52^{g}	46.71±2.43^{bg}	47.65±0.37^{abg}	43.69±0.82^{abcg}	42.84±0.78^{abcdg}	37.74±3.90^{abcdfg}	39.43±3.47^{abcdefg}	32.35±2.73^{abcdefgh}	22.93±0.74^{bcdefgh}	3.20±0.67^{i}	2.57±2.44^{i}	0.29±0.09^{i}	0.23±0.14i
Maestà	39.01±0.80	19.85±1.76^{g}	37.87±3.13^{f}	53.69±2.11^{af}	47.50±1.19^{abf}	41.49±1.24^{abdf}	41.55±1.46^{abcdf}	39.13±0.36^{abcdef}	35.53±1.96^{bcdef}	31.10±1.34^{cdefg}	20.18±3.10^{g}	1.96±0.75^{h}	0.50±0.08^{h}	0.34±0.16^{h}	0.20±0.07^{h}
Iride	43.16±0.79	20.64±1.15^{e}	44.48±1.92^{c}	56.96±0.17^{ac}	54.46±0.02^{abc}	43.09±0.31^{abcf}	40.24±3.21^{bcfg}	40.58±2.52^{bcfg}	44.47±4.40^{abcdfg}	32.13±2.70^{cdfg}	15.72±0.36^{e}	1.38±0.10^{h}	0.55±0.05^{h}	0.41±0.02^{h}	0.28±0.11^{h}
